# Addressing the Health Needs of Underserved Populations Through Public Contribution: Prioritisation and Development of a Peer Support Intervention for Sexual and Gender Minority Forced Migrants

**DOI:** 10.1111/hex.70277

**Published:** 2025-05-06

**Authors:** Tommy Carlsson, Rogers Kissiti, Maria Jirwe, Elisabet Mattsson, Louise von Essen, Maria Gottvall

**Affiliations:** ^1^ The Department of Health Sciences The Swedish Red Cross University Stockholm Sweden; ^2^ CIRCLE ‐ Complex Intervention Research in Health and Care, The Department of Women's and Children's Health Uppsala University Uppsala Sweden; ^3^ The Division of Nursing, Department of Neurobiology, Care Sciences and Society Karolinska Institute Stockholm Sweden; ^4^ The Department of Health Care Sciences Marie Cederschiöld University Stockholm Sweden

**Keywords:** forced migrants, patient and public involvement, priority setting, sexual and gender minorities

## Abstract

**Introduction:**

The health of underserved populations, including sexual and gender minority forced migrants, is a pressing global concern. Public contribution in research has the potential to enhance prioritisation and aid in intervention development, but has been criticised due to a lack of sufficient diversity and engagement with underserved populations.

**Methods:**

The core research team conducted eight workshops together with eight experts by lived experience to prioritise and guide future peer support intervention research. Activities included brainstorming, pathway mappings, ranking procedures, storytelling exercises, photovoice sessions and individual open‐ended writing sessions. Open‐ended reflective meetings and manifest content analysis of material, as well as documentation, guided the progress towards final results.

**Results:**

Peer support was identified as an intervention with the potential to reduce mental health burdens, enhance the capacity to integrate into society and provide access to basic needs. Peer support interventions aiming to reduce health inequities by promoting language proficiency and employment attainment were identified as prioritised areas. A range of considerations and barriers regarding the modality of interventions, the training of peer supporters and recruitment strategies needs further examination in research.

**Conclusions:**

Our findings illustrate the importance of public contribution when planning research addressing support for underserved and marginalised populations. Public contribution efforts targeting underserved populations such as ours will help researchers gain an in‐depth understanding of prioritised research questions and pragmatic study procedures. In regard to research for sexual and gender minority forced migrants, we recommend prioritisation of intervention development that promotes mental health and reduces loneliness through support from peers in group settings and from peer mentors, informational support and capacity‐building.

**Patient or Public Contribution:**

Representatives acting as experts by lived experience contributed as research partners throughout the procedures and workshops.

## 1 Introduction

Public contribution in research has been acknowledged as a method that can democratise research, reduce research waste and prioritise research in line with the needs and preferences of service users [1, 2, 3]. There is a recognised need to develop relevant and tailored interventions in close collaboration with target populations, especially those who are underserved in research [4]. However, research utilising public contributions has been criticised for lacking sufficient diversity among those collaborating [5], and few efforts have been made to engage with underserved populations to tailor interventions in health and social services [6, 7, 8]. This calls attention to the risk of continuing to overlook the needs among seldom heard and marginalised populations [9, 10]. Research still lacks a comprehensive understanding of how to engage with contributors in meaningful ways that take diversity in the general population as well as the specific needs of underserved populations into consideration [11]. While involving excluded groups in society through prioritisation and development can result in relevant and acceptable support interventions [12], there is a lack of such research conducted in collaboration with forced migrant minority populations.

With over 100 million forcibly displaced persons worldwide [13], the health and well‐being of forced migrants are a pressing global concern. In several countries, sexual and gender minority individuals are exposed to severe victimisation and structural oppression [14]. These persons recount powerful stories of violence and abuse enacted by a wide range of perpetrators, including beatings and assaults, psychological abuse, harassment, blackmailing, threats and sexual violence [15]. Faced with grave danger and persecution, many have no other option than to flee and seek refuge in another country. However, their journeys are far from safe, as many are exposed to violence and disadvantages based on a double minority status [16]. After arrival in the host country, they face numerous challenges and are at risk of experiencing psychological distress [17]. Sexual and gender minority forced migrants encounter intersectional discrimination and violence enacted by a range of perpetrators, including diaspora co‐ethnic community members, others within the sexual and gender minority community, and the mainstream society [18]. During transit and resettlement, these migrants experience severe levels of depression, anxiety and post‐traumatic stress [16, 17]. Loneliness and social exclusion are prominent concerns, further impacting health and well‐being [16, 17, 19]. Despite the dangers and adversities encountered throughout their migration trajectories, these persons appreciate the right to live authentically after resettlement, showing significant resilience and utilising a range of resources for support [17, 20].

Structural disadvantages and unmet health needs contribute to health inequities and impact forced migrants' ability to recover from pre‐migration trauma [21]. Host countries have a responsibility to support people who seek asylum, including those seeking asylum based on sexual and gender minority status [22]. Although non‐governmental organisations and volunteers offer support for these migrants [23], research efforts to develop tailored interventions addressing the needs within the population are lacking. The overall aim of this study was to prioritise and guide future research developing and testing tailored peer support interventions, through the collaboration with experts by lived experience as sexual and gender minority forced migrants. Four research questions were addressed, focusing on future research offering support interventions for sexual and gender minority forced migrants:

1. Which psychosocial challenges should be prioritised?
2. Which recruitment strategies should be utilised to reach potential participants in research and when delivering support interventions?
3. What outcomes are relevant and should be prioritised when evaluating peer support interventions?
4. How should peer support interventions be structured and delivered?

## 2 Materials and Methods

### 2.1 Study Design

The overall study design was a collaborative approach in which researchers engaged in collaboration with experts by lived experience (hereafter referred to as ‘experts’) to prioritise and guide future research. A community‐based participatory action research approach was applied, in which knowledge is generated through a process that places people with lived experience in the centre as experts [24]. Throughout the process, experts were considered as research partners in the team and were employed as research assistants at the university where the project was conducted. The term experts by lived experience was chosen to emphasise the expertise and knowledge based on unique and personal experiences, while also underscoring their role as active partners in shaping the research agenda. We acknowledge that other terms (e.g., public contributors or research partners) may be used for similar roles and tasks when conducting public involvement in research.

The core research team consisted of one coordinator who is an expert by lived experience and two Swedish‐born health professional researchers (PhD and registered nurse‐midwives). This core team planned and participated in consecutive workshops together with experts. Experts were employed as research assistants at the university and were paid a salary for their work, depending on the number of workshops they attended. All experts were free to decide which workshops they attended and did not have to state any reason for non‐attendance.

### 2.2 Sampling and Workshop Attendants

A total of eight experts were recruited through purposeful, snowball and advertisement sampling to reach diversity, utilising the established networks of the research team. The experts represented diverse sexual orientations and gender identities (e.g., trans/gender non‐conforming persons, lesbian women and gay men). All were adults in their 20s, 30s and 40s, originating from countries in Africa, Asia and Europe. Additionally, two consultants (licensed clinical psychologists, reimbursed for their work) were involved in targeted consultations about clinical considerations [25].

### 2.3 Workshop Sessions

A series of eight sequential workshops were conducted during 5 months in 2023 with the purpose of addressing the research questions. The number of workshops was determined through the research questions. The duration of each workshop was 5 h. All workshops began with a joint lunch, and refreshments were served during breaks. Focus areas for each workshop were inspired by the Template for Intervention Description and Replication (TIDieR) checklist [26]. Having determined the focus areas, the specific activities conducted during the workshops were inspired by the Iriss co‐production project planner [27]. The workshops were exploratory and inductive, in which experts and researchers engaged in mutual discussions. The core research team moderated discussions and asked follow‐up questions. Please see Table 1 and Appendix 1 for a presentation of the focus areas and activities of each of the eight workshops.

**Table 1:** Presentation of the focus areas and activities in each workshop (W) with experts by lived experience (ELE), researchers (R) and clinical consultants (CC).

W	Attendants	Focus area	Activities, groups (*n*)
1	ELE (*n* = 9), R (*n* = 2), CC (*n* = 1)	Psychosocial challenges	Brainstorming and pathway mapping, 2
		Psychosocial support	Brainstorming and pathway mapping, 2
2	ELE (*n* = 9), R (*n* = 1), CC (*n* = 1)	Prioritising psychosocial challenges	Individual ranking procedure, 1
		Finding and structure of peer support	Storytelling exercises, 3
3	ELE (*n* = 7), R (*n* = 2)	Meaning of social support	Photovoice session, 1
		Impact of peer support	Storytelling exercises, 3
4	ELE (*n* = 6), R (*n* = 2)	Feedback on drafts based on the findings of previous workshops	Each ELE provided written comments followed by joint discussions, 1
5	ELE (*n* = 2), R (*n* = 3), CC (*n* = 1)	Feedback on drafts, focused on clinical considerations	Discussion of co‐produced material from previous workshops, 1
6	ELE (*n* = 7), R (*n* = 2)	Prerequisites and criteria for a peer support intervention	Discussions with a focus on the criteria, delivery and training of supporters, 3
			Preparation of a fictional newspaper headline about intervention, 3
7	ELE (*n* = 8), R (*n* = 2)	Activities in a peer support intervention	Discussions about benefits, structure and considerations of activities, 3
8	ELE (*n* = 5), R (*n* = 1)	Activities in a peer support intervention	Individual creative writing followed by joint discussions, 1
			Individual ranking procedure, 1

Field notes were taken by members of the core research team. No sensitive information about individual experts was collected during workshops. The co‐produced material was stored in locked storage according to local routines at the host university. Following each workshop, the core research team had open‐ended reflective meetings, collaborated to summarise the findings and planned for subsequent workshops. After concluding the final workshop, the core research team held joint meetings to summarise the overall findings, scrutinise the work and draft a report presenting the findings. The report was formatted according to the Guidance for Reporting Involvement of Patients and the Public (GRIPP2) checklist, please see Appendix 2 [28].

## 3 Results

### 3.1 Which Psychosocial Challenges Should be Prioritised in Research?

The experts called attention to numerous psychosocial challenges experienced by the target population while residing in Sweden, structured in four categories with eight prioritised areas (Table 2). A range of mental health burdens were considered highly prioritised, with loneliness and social exclusion being the most prioritised areas for future research to address. According to the experts, loneliness can be exacerbated by difficulties transitioning into the new society, living in asylum accommodations and encountering multi‐layered discrimination in society. Additionally, experts pointed out difficulties accessing health and social services, a lack of competence among those involved in the asylum process, and challenges finding housing and employment. Workshops highlighted the need for a range of support interventions, structured into four categories (Table 3). Support from peers in group settings and from peer mentors, informational support and capacity‐building to secure employment and safe housing were emphasised as prioritised interventions in need of further attention.

**Table 2:** Highlighted post‐migration challenges and rankings of prioritisation in research (some challenges received the same ranking).

Category	Rank	Challenge
Mental health burdens	1	Loneliness and social exclusion
	2	Depression, sadness and hopelessness
	3	Post‐traumatic stress
	4	Stress and anxiety
	5	Self‐blame, self‐stigma and internalised homophobia
	6	Suicidality
	7	Confusion
		Fears
Health and social services	1	Difficulties navigating the health and social service system
		Lack of access or too long waiting time for services
	2	Interpreters not qualified in sexual and gender minorities
	3	High costs associated with health services
	4	Discrimination in services
Migration process	1	Lack of competence and discrimination in asylum accommodations
		Lack of competence among interpreters
		Lawyers/caseworkers lack competence in sexual and gender minorities
		Social exclusion when living in camps
	2	Long waiting time for asylum decisions
	3	Insufficient protection when living in camps
		Insufficient support from social services
		Lack of information or updates about the migration process
	4	Discrimination by other migrants at camps
Psychosocial challenges encountered in society	1	Difficulties securing housing
		Financial instability and difficulties securing employment
	2	Structural discrimination of sexuality, gender, racism, stigma and phobia
	3	Difficulties finding a partner, family and friends
		Exclusion from social areas or gatherings
		Mandated housing arrangements with non‐selected cohabitants
	4	Difficulties with paperwork and bureaucracy
		Language barriers
	5	Lack of protection enforced by the authorities
	6	Sexual exploitation and sex work

**Table 3:** Highlighted suggestions for interventions providing post‐migration support.

Category	Support structure
Professional support	Additional support from social workers
	Assistance in navigating the healthcare system
	Improved access to health services
	Interventions containing information
	Enhancing competence in health and social services
	Mental health support from professionals
Social/peer support	Group‐based peer support activities
	Individual peer mentorship
	Gatherings to practice language skills
Organisational or societal development	Ensure access to lawyers competent in sexual and gender minorities
	Ensure sufficient competence of workers at camps
	Increase funds for non‐governmental organisations
	Promote opportunities for securing employment
Leisure and recreational activities	Dance, music and entertainment
	Games and sports
	Meditation
	Writing and reading

### 3.2 Which Recruitment Strategies Should be Utilised to Reach Potential Participants, in Research and When Delivering Support Interventions?

Workshop activities revealed routes through which sexual and gender minority forced migrants could be approached for participation in research, including in‐person (e.g., via non‐governmental organisations and entertainment venues) and online (e.g., via social media platforms and dating sites) methods. Mainly, these routes involved community‐based convenience or purposeful sampling through advertisements and snowball sampling through word‐of‐mouth. Experts also raised potential barriers for recruitment, which encompassed language barriers, issues of trust, geographical distances and financial constraints.

### 3.3 What Outcomes Are Relevant and Should be Prioritised When Evaluating Peer Support Interventions?

The experts highlighted a range of possible outcomes and benefits of participating in peer support interventions from a general perspective. Peer support was considered to have a potential to improve the following mental health and well‐being outcomes: (1) feeling accepted and included, (2) loneliness, (3) emotional relief, (4) empowerment, (5) self‐acceptance, (6) self‐esteem, (7) happiness and hopefulness, (8) sleep quality, (9) feeling stigmatised, (10) symptoms of post‐traumatic stress, (11) quality of life, (12) sense of safety and (13) experiencing mutual trust. Experts also believed that hospital admissions could be reduced when participating in a peer support intervention. A potential positive effect on information and capacity development outcomes was also expressed, including: (1) new opportunities for societal establishment (e.g., attaining education and employment, dating, language skills, expanding legal circumstances and developing social networking) and (2) enhanced knowledge (e.g., learning about health and health services, non‐governmental organisations, integration issues, host country society and societal norms, sexuality, gender identity and expression, and social services). Additionally, peer support was considered to have a potential positive impact on access to basic subsistence (e.g., improving access to clothing, food, housing, the Internet, physical activities, organisation memberships and transportation).

In addition to the potential outcomes leading to health improvements, experts also highlighted 10 potential risks of peer support interventions. These included: (1) not meeting expectations among participants, (2) participants and supporters not being resilient enough to cope with the information exchanged, (3) unequal service provision and not being able to accommodate all needs, (4) some participants may feel insecure when interacting with peers, (5) lack of respect among participants, (6) gossip and disclosure of sensitive information, (7) racism towards some participants, (8) envy among participants, (9) trust issues and (10) inappropriate information provided during sessions.

### 3.4 How Should Peer Support Interventions for These Migrants be Structured and Delivered?

Potential advantages and disadvantages related to the modality of peer support and the need for training of peer supporters were summarised (Table 4). Overarchingly, in‐person peer support was considered to be associated with more advantages and fewer disadvantages than digital support. In‐person support led by trained supporters was considered a safer and confidential option. Identified criteria for trained peer supporters included being able to show compassion through lived experience, having adequate communication skills and adhering to work ethics. Based on discussions and ranking procedures, seven activities within peer support interventions were identified, the most prioritised being capacity‐development in language skills and securing employment, followed in descending order by activities focusing on mental health and relaxation, cooking, dating, and games and sports. Please see Appendix 3 for an expanded presentation of the identified activities. Requirements for all types of peer support interventions were: ensuring safe settings at an accessible place when arranging in‐person activities, participants have access to technical equipment when arranging digital activities and participants and supporters have basic levels of language proficiency and digital literacy when needed.

**Table 4:** Discussed advantages, disadvantages and risks in ways to provide peer support.

How to provide support		Advantages	Disadvantages and risks
Modality	In‐person	Builds a large support network	Inaccessibility
		Cost‐effectiveness	Challenging for some to open up
		Enhances the reliability of information	Expenses (e.g., supporters/employees)
		Involves interactivity between peers	Bad intentions among participants
		Opportunity for nonverbal communication	
		Potential to feel safe	
		Promotes deep peer relationships	
	Digital	High accessibility	Equipment requirements
		Can involve anonymity	Limited opportunity for deep relationships
		Convenient and time‐saving	Missing nonverbal communication
			Requires digital literacy
			Risk of technical malfunctions
			Risks related to security and privacy
			Uncertainties about identity and intentions
Training of peer supporters	Non‐trained	Cost‐effectiveness	Confidentiality issues
		Pragmatic recruitment of supporters	Insensitivity towards gender identity
		Committed supporters	Inaccurate information
			Irresponsibility and unprofessionalism
			Lack of experience and qualifications
			Low quality of support and ineffectiveness
	Trained	Accurate and reliable information	High expenses
		Competent and disciplined supporters	Need for qualified staffing
		Confidentiality ensured	Power imbalance and misuse
		High intervention fidelity	Time needed to train supporters
		Professionalism and respectfulness	Too enthusiastic supporters

## 4 Discussion

### 4.1 Principal Findings

The overall aim of this study was to prioritise and guide future research developing and testing tailored peer support interventions. Given the higher morbidity and mortality in less privileged groups within society [29, 30], there is a pressing need to undertake targeted actions that counteract socially produced inequities impacting underserved populations [4, 12]. We engaged in collaboration with experts by lived experience through public contribution in research, which has the potential to increase the relevance and quality of research and thus can help in reducing research waste [1, 4]. Few efforts have been made to engage with underserved populations to co‐produce research [6, 8], and there is an acknowledged lack of diversity within prior research utilising public involvement [9, 10]. Our study brings new insights into collaboration to prioritise and guide future research addressing the health and well‐being of sexual and gender minority forced migrants, which is an underserved population. Based on our findings, we recommend that researchers prioritise intervention development that involves support from peers in group settings and from peer mentors, informational support and capacity‐building.

Our study focused on tailored peer support interventions for sexual and gender minority forced migrants. Despite significant health burdens and societal disadvantages in the host country [15, 16, 17], few research efforts have been made to develop and evaluate support interventions for this target population [31]. Our study complements prior qualitative studies about support needs and preferences, providing valuable insights about prioritisation for future peer support research. In line with our findings, previous research illustrates that sexual and gender minority forced migrants desire and engage in peer support following their arrival [17]. As indicated by our findings, peer support can involve important emotional, informational and practical support between laypersons sharing similar experiences or identities [32, 33]. Peer support has been suggested to be an effective intervention for underserved populations [34] and can involve various beneficial effects on health and well‐being both in sexual and gender minority populations [31, 35], as well as among forced migrants [36, 37]. We identified a range of potential positive effects, the highest ranked among our experts being reduced loneliness and social exclusion. Researchers conducting peer support intervention research for the target population should draw inspiration from our list of outcomes when considering the evaluation of their intervention. We recommend more research on peer support interventions for this population.

From a wider perspective, the recruitment strategies suggested by our experts may help researchers when planning research addressing the health and well‐being of underserved and marginalised populations. Research addressing underserved and marginalised populations deserves special methodological considerations, including how to achieve successful participant recruitment [38]. Studies have highlighted various barriers to participation in research among sexual and gender minority individuals and forced migrants, including logistical challenges, potential mistrust, lack of awareness, language barriers, and concerns about confidentiality and financial incentives [39, 40]. This study revealed community‐based recruitment as a potentially pragmatic approach when conducting research to develop and test support interventions for sexual and gender minority forced migrants. Community‐based recruitment has been highlighted as a potential strategy for effective recruitment of sexual and gender minority individuals [41]. More research is needed about the feasibility of different recruitment strategies to successfully reach and enrol seldom heard groups, and specifically, sexual and gender minority forced migrants.

### 4.2 Methodological Considerations

There are methodological limitations that readers need to consider when interpreting our findings. We collaborated with eight experts by lived experience throughout eight workshops, to guide and plan future research addressing the health and well‐being of sexual and gender minority forced migrants. The experts were recruited using purposeful sampling and utilising the networks among the researchers and project collaborators. While the experts represented diverse identities and backgrounds, we acknowledge that the findings should be viewed as exploratory, and we encourage additional efforts to plan and prioritise future research. We believe that we reached diversity among our experts and that the exploratory approach applied during the workshops increased our opportunities to reach novel findings. Nevertheless, utilising eight experts is a limitation. We encourage more research utilising public involvement of sexual and gender minority forced migrants.

Some challenges were encountered when conducting this study process. Significant time and effort were needed to plan the workshops, and while several people were involved in the planning, it was nevertheless challenging to plan accordingly. Researchers who want to undertake similar studies need to ensure that sufficient resources are allocated in the planning phase. Moreover, the workshops required substantial administrative tasks like arranging refreshments, locales, materials and communication with the human resources department. This needs careful consideration when planning similar public contribution efforts. Another crucial aspect was the need for funding to conduct the research and reimburse the experts by lived experience. Our public contribution efforts were highly successful in helping us plan and prioritise future research. We encourage research funders to continue supporting research utilising public contributions to prioritise and co‐design future research.

## 5 Conclusions

Our findings illustrate the importance of public contribution when planning research that addresses support for underserved and marginalised populations. The procedures utilised herein aided in the prioritisation of outcomes, identification of barriers for successful participant recruitment and recognition of potential activities within future interventions addressing the health and well‐being of sexual and gender minority forced migrants. Public contribution efforts targeting underserved populations such as ours will help researchers gain an in‐depth understanding of prioritised research questions and pragmatic study procedures. In regard to research on sexual and gender minority forced migrants, we recommend the prioritisation of intervention development that involves support from peers in group settings and from peer mentors, informational support and capacity‐building. These interventions have the potential to promote mental health and reduce loneliness.

## Author Contributions

**Tommy Carlsson:** conceptualisation (lead), data curation (equal), formal analysis (equal), funding acquisition (lead), methodology (equal), project administration (equal), supervision (lead), writing – original draft preparation (lead). **Rogers Kissiti:** data curation (equal), methodology (supporting), project administration (equal), writing – review and editing (supporting). **Maria Jirwe:** conceptualisation (supporting), funding acquisition (supporting), writing – review and editing (supporting). **Elisabet Mattsson:** conceptualisation (supporting), funding acquisition (supporting), writing – review and editing (supporting). **Louise von Essen:** conceptualisation (supporting), funding acquisition (supporting), writing – review and editing (supporting). **Maria Gottvall:** conceptualisation (lead), data curation (equal), formal analysis (equal), funding acquisition (supporting), methodology (equal), project administration (equal), supervision (supporting), writing – original draft preparation (supporting).

## Ethics Statement

The Swedish Ethical Review Authority provided a waiver based on the research not falling under the provisions of the Ethical Review Act in Sweden (Application number 2023‐00015‐02). This study should be considered as public involvement/contribution in research. Experts by lived experience who took part in our workshops were not research participants and were employed as research assistants throughout the process.

## Conflicts of Interest

The authors declare no conflicts of interest.

## Supporting information

Appendix 1. Descriptions of the eight workshops (W) in which researchers (R) collaborated with experts by lived experience (ELE) and clinical consultants (CC).

Appendix 2. GRIPP2 short form.

Appendix 3. Identified peer support activities.

## Data Availability

The data that support the findings are available on request from the corresponding author. The data are not publicly available due to privacy or ethical restrictions.
