# Supplementary material for: Addressing the Health Needs of Underserved Populations Through Public Contribution: Prioritisation and Development of a Peer Support Intervention for Sexual and Gender Minority Forced Migrants
Source: Health Expect. 2025 May 6;28(3):e70277. doi: 10.1111/hex.70277 (PMC12053740; doi:10.1111/hex.70277)
Supplement: Supplementary file 1 — Appendix 1. Descriptions of the eight workshops (W) in which researchers (R) collaborated with experts by lived experience (ELE) and clinical consultants (CC). [file HEX-28-e70277-s002.docx]

**Appendix 1.** Descriptions of the eight workshops (W) in which researchers (R) collaborated with experts by lived experience (ELE) and clinical consultants (CC).

| **W** | **Attendants** | **Focus area** | **Description of activities during the workshop** | **Co-produced material** | **Findings** |
| --- | --- | --- | --- | --- | --- |
| 1 | ELE (n=9)  R (n=2)  CC (n=1) | Psychosocial challenges and psychosocial support | **Brainstorming and pathway mapping**  The experts were divided into two groups and asked to discuss and summarize post-migration: (1) psychosocial challenges and (2) desired and appreciated psychosocial support. Pathway mappings were produced illustrating the trajectory for migrants. The activity was followed by a joint discussion among all experts, in which each group was asked to present their findings to all team members. | Annotations and drawings written on whiteboards and large papers; field notes | Several challenges were highlighted, structured into four categories: (1) mental health burdens, (2) health- and social services, (3) the migration process, and (4) structural psychosocial challenges in society. Pathway mappings highlighted various challenges throughout the migration and resettlement trajectory; some remaining throughout the process and considered highly impactful (e.g., financial instability, housing, and employment).  Workshops highlighted the need for interventions structured into four categories: (1) professional support, (2) social/peer support, (3) organizational or societal development, and (4) leisure and recreational activities. Informal support from a peer who helps and understands your situation was highlighted as particularly promising. Experts discussed interventions providing information about a range of topics and highlighted the need for interventions improving the situation of forced migrants with diverse SOGIE living in asylum accommodations. Interventions that enhance opportunities to secure employment were highlighted. |
| 2 | ELE (n=9)  R (n=1)  CC (n=1) | Prioritizing psychosocial challenges | **Individual ranking procedure**  Each expert was asked to individually rank the challenges identified in workshop 1 by providing a maximum of three stars for challenges within each of the identified categories: (1) mental health burdens, (2) health- and social services, (3) migration process, and (4) structural psychosocial challenges in society. The session was concluded with a discussion in which all experts were asked to share something about their reasonings behind their rankings. | Individual quantitative rankings as number of stars provided with pen and paper; field notes | The ranking procedure for prioritization in research revealed seven prioritized areas: (1) loneliness and social exclusion, (2) difficulties knowing where to get help and navigating the health- and social service system, (3) lack of competence among workers, interpreters, and lawyers/caseworkers, (4) social exclusion when living in camps, (5) difficulties securing housing, (6) financial instability, and (7) difficulties securing employment. Loneliness and social exclusion were provided the most stars out of all identified challenges. |
|  |  | Finding and structure of peer support | **Storytelling exercise**  The experts were divided into three groups and prompted to write about a fictional character arriving in Sweden and looking for peer support. A prepared document contained open-ended questions guiding the storytelling, including questions about where the character looked for peer support, how they interacted with peers, and what attributes the support had. The session was concluded with a joint discussion about the stories. | Written documents of a story about a fictional character provided with pen and paper | Stories and discussions revealed routes through which forced migrants with diverse SOGIE could be approached for research-related purposes, including in-person (e.g., activities via non-governmental organizations and entertainment venues) and online methods (e.g., social media and dating sites). Mainly, these routes involve community-based convenience sampling through advertisements and snowball sampling through word-of-mouth. Potential barriers to peer support included language barriers, trust issues, distances, and financial constraints. |
| 3 | ELE (n=7)  R (n=2) | Meaning of social support | **Photovoice session**  Each expert was asked to provide photographs illustrating what peer support can mean for forced migrants with diverse SOGIE. The activity was concluded with a joint session in which experts looked at all photographs and had joint discussions | Photographs; field notes | A range of potential benefits of social/peer support were highlighted, structured into three categories: (1) mental health and wellbeing, (2) information and capacity development, and (3) access to basic subsistence.  Potential risks and adverse events when exchanging peer support were highlighted, structured into ten categories: (1) unsuccessfully meeting expectations among participants, (2) participants and supporters not being resilient enough to cope with the information exchanged during peer support activities, (3) inequality in service provision and not being able to accommodate all needs among participants, (4) some participants feeling insecure when interacting with peers, (5) lack of respect among participants, (6) gossip and disclosure of sensitive information, (7) racism towards some participants, (8) jealousy among participants, (9) trust issues, and (10) inappropriate information provided during sessions. |
|  |  | Impact of peer support | **Storytelling exercise**  The experts were divided into three groups and prompted to continue writing the story about the fictional character initialized in workshop 2. A prepared document contained open-ended questions guiding the storytelling, including questions about the benefits and potential risks or disadvantages when engaging in peer support. The session was concluded with a joint discussion about the stories. | Written documents of a story about a fictional character |  |
| 4 | ELE (n=6)  R (n=2) | Feedback on drafts | **Feedback session**  All experts were asked to individually read drafts prepared by the core research team, based the findings of the previous workshops. The drafts addressed state-of-the-art, purpose, significance, and methodology for an intended research project investigating the effects of peer support among migrants with diverse SOGIE. In between reading the different sections, the experts engaged in joint discussions. | Written comments; field notes | Experts highlighted the need to further illustrate the challenges and potential benefits of a peer support intervention. Specific comments were provided which helped by guiding the work moving forward. |
| 5 | ELE (n=2)  R (n=3)  CC (n=1) | Feedback on drafts, clinical considerations | **Feedback session**  All attendees were asked to prepare by reading the drafts as in workshop 4. Attendants engaged in joint discussions together with a consultant (specialist clinical psychologist) focusing on clinical considerations when providing peer support interventions. | Written comments; field notes | Discussions highlighted the need for clear structures in which participants can be referred to trauma-informed services when needed. Specific comments were provided which helped by guiding the work moving forward. |
| 6 | ELE (n=7)  R (n=2) | Prerequisites and criteria for a peer support intervention | **Focused discussions in smaller groups**  The experts were divided into three groups and asked to have a focused discussion in which three topics were covered: (1) advantages and disadvantages of in-person and digital delivery of peer support interventions, (2) advantages and disadvantages of training or non-training of supporters in peer support interventions, and (3) criteria for trained supporters in peer support interventions. Each group summarized their discussions as bullet points. The session was concluded with a joint discussion. | Documents with written bullet points; field notes | While in-person peer support was considered to involve reliability and enhanced communication opportunities, digital alternatives were considered accessible and suited for those who desire anonymity. In-person support were considered to require refreshments for participants and accessible safe locales. Drawbacks of digital support were requirements of technical equipment and digital literacy, requiring access to staffing providing technical assistance when needed, and risks of breaking confidentiality, requiring dependable and safe technical solutions.  While having trained peer supporters was considered to involve quality and competence when delivering support, non-training was considered a pragmatic approach involving committed supporters.  When discussing the characteristics and criteria for trained peer supporters, the following categories were addressed as important: (1) being able to show compassion through lived experience, (2) having adequate communication skills, and (3) adhering to work ethics. |
|  |  |  | **Tomorrow’s headlines session**  The three groups were asked to produce a fictional newspaper headline describing a peer support intervention for forced migrants with diverse SOGIE. Experts were prompted to think about the impact of a research intervention and be creative when producing the document. The session was concluded with a joint discussion in which each group presented their newspaper document. | Newspaper documents; field notes | News headlines illustrated the impact peer support intervention could have.  Addressed potential benefits on a societal level included societal visibility, awareness, and equality.  Addressed potential benefits on an individual level included improved mental health, reduced suicidality, enhanced empowerment, reduced symptoms of post-traumatic stress, and reduced loneliness. |
| 7 | ELE (n=8)  R (n=2) | Intra-intervention activities in a peer support intervention | **World café session**  The experts were divided into three groups and asked to rotate in a room with different stations in which one of the following activities embedded in a peer support intervention were discussed, identified through the previous workshops: (1) capacity-development in language skills, (2) capacity-development in securing employment, (3) mental health, relaxation, and mindfulness, (4) cooking together, (5) dating, and (6) games and sports. With pen and paper, the groups summarized the potential benefits, needed structure, and considerations for each activity as bullet points for each activity, respectively. The session was concluded with a joint discussion. | Documents with written bullet points; field notes | World café discussions and creative writing revealed different considerations and perspectives on the activities.  Capacity-development in language skills: Group-based or individual sessions with language teachers were considered to have benefits for societal integration, confidence, self-esteem, anxiety, stress, communication skills, and socialization. It was considered important that such an intervention ensure that participants feel included and welcome when having diverse SOGIE, regardless of their language proficiency.  Capacity-development in securing employment: Group-based activities or individual sessions together with expertise were considered to have benefits for empowerment, improved confidence, and reduced anxiety. Further, it was considered to motivate you to seek employment and provide knowledge how to establish yourself in the host country society (e.g., writing a CV). It was considered challenging for migrants to find reliable employment in line with their qualifications without a risk of exploitation.  Mental health, relaxation, and mindfulness: Activities with instructors and learning about how to maintain mental health was considered important for forced migrants who have faced oppression, and to have benefits for their overall mental health, including improved ability to recognize unconscious negativity, engage in self-reflection, sleep, and focus. It was also addressed to enhance communication skills, stimulate empathy and kindness, and enhancing awareness of others. Becoming relaxed when partaking in relaxation and mindfulness activities was considered to reduce loneliness, counteract negative thinking, enhance productivity, and be empowering.  Cooking together: Group-based sessions were considered to have benefits for social networking and bonding, promotion of health and wellbeing, mental stress, and increased knowledge about healthy food and culture. Cooking was addressed as an enjoyable activity that brings people together, creates bonds, establishes trust, promotes socialization, and provides a healthy meal to those in need.  Dating: Group-based activities and creating a dating site were considered to have potential benefits for finding meaningful and strong relationships, which can lead to improved overall mental health, self-esteem, relaxation, sexual health, societal integration, and reduced loneliness. It was considered challenging for forced migrants with diverse SOGIE to date and find a partner, involving a risk of encountering abuse, unhealthy habits, contracting sexually transmitted infections, and feeling disappointed. A related suggested intervention involved health education about consent, dating, and safe sex practices.  Games and sports: Group-based activities were considered to have benefits for physical and mental health, including sleep, mental focus, stress, anxiety, and happiness. Participating in games and sports were addressed as bringing people together and helping them find meaningful company, while also promoting them to gain healthy habits and feel freedom, pleasure, optimism, and creativeness.  Common requirements for all types of peer support interventions were ensuring safe settings at an accessible locale when arranging in-person activities, accessibility to needed technical equipment when arranging digital activities, and that participants had basic levels of language proficiency/digital literacy to communicate with each other. |
| 8 | ELE (n=5)  R (n=1) | Intra-intervention activities in a peer support intervention | **Creative writing session**  The experts were asked to provide written statements about each of the identified activities from workshop 7, through a session of individual creative open-ended writing. After having written about one of the activities, the experts engaged in a joint discussion about the reflections, before moving on to the next activity. | Written statements of each identified activity; field notes |  |
|  |  |  | **Individual ranking procedure**  Following the creative writing session, all experts were asked to rank the priority of each of the identified activities, by providing a maximum of five stars (1 star: no priority, 5 stars: highest priority). | Individual quantitative rankings as number of stars | In the final ranking procedure, the experts ranked capacity-development as having the highest priority by providing it the most stars in total. |
