# Supplementary material for: Addressing the Health Needs of Underserved Populations Through Public Contribution: Prioritisation and Development of a Peer Support Intervention for Sexual and Gender Minority Forced Migrants
Source: Health Expect. 2025 May 6;28(3):e70277. doi: 10.1111/hex.70277 (PMC12053740; doi:10.1111/hex.70277)
Supplement: Supplementary file 2 — Appendix 2. GRIPP2 short form. [file HEX-28-e70277-s001.docx]

**Appendix 2.** GRIPP2 short form (Staniszewska et al., 2017).

| **Section and topic** | **Item** | **Reported on page No** |
| --- | --- | --- |
| 1: Aim | Report the aim of PPI in the study | 3 |
| 2: Methods | Provide a clear description of the methods used for PPI in the study | 4-5 |
| 3: Study results | Outcomes—Report the results of PPI in the study, including both positive and negative outcomes | 5-7 |
| 4: Discussion and conclusions | Outcomes—Comment on the extent to which PPI influenced the study overall. Describe positive and negative effects | 7-10 |
| 5: Reflections/critical perspective | Comment critically on the study, reflecting on the things that went well and those that did not, so others can learn from this experience | 8-9 |

**References**

Staniszewska, S., Brett, J., Simera, I. et al. GRIPP2 reporting checklists: tools to improve reporting of patient and public involvement in research. Res Involv Engagem 3, 13 (2017). https://doi.org/10.1186/s40900-017-0062-2
