# Supplementary material for: Addressing the Health Needs of Underserved Populations Through Public Contribution: Prioritisation and Development of a Peer Support Intervention for Sexual and Gender Minority Forced Migrants
Source: Health Expect. 2025 May 6;28(3):e70277. doi: 10.1111/hex.70277 (PMC12053740; doi:10.1111/hex.70277)
Supplement: Supplementary file 3 — Appendix 3. Identified peer support activities. [file HEX-28-e70277-s003.docx]

Appendix 3. Identified peer support activities.

| **Rank** | **Activity** | **Summary** |
| --- | --- | --- |
| 1 | Capacity-development in language skills | Group-based or individual sessions with language teachers were considered to have benefits for societal integration, confidence, self-esteem, anxiety, stress, communication skills, and socialization. It was considered important that such an intervention ensure that participants with diverse SOGIE feel included and welcomed regardless of their language proficiency. |
| 2 | Capacity-development in securing employment | Group-based activities or individual sessions together with expertise were considered to have benefits for empowerment, improved confidence, and reduced anxiety. Further, it was considered to motivate you to seek employment and provide knowledge how to establish yourself in the host country society (e.g., writing a CV). It was considered challenging for migrants to find reliable employment in line with their qualifications without a risk of exploitation. |
| 3 | Mental health, relaxation, and mindfulness | Activities with instructors and learning about how to maintain mental health was considered important for forced migrants who have faced oppression, and to have benefits for their overall mental health, including improved ability to recognize unconscious negativity, engage in self-reflection, sleep, and focus. It was also addressed to enhance communication skills, stimulate empathy and kindness, and enhancing awareness of others. Becoming relaxed when partaking in relaxation and mindfulness activities was considered to reduce loneliness, counteract negative thinking, enhance productivity, and be empowering. |
| 4 | Cooking | Group-based sessions were considered to have benefits for social networking and bonding, promotion of health and wellbeing, mental stress, and increased knowledge about healthy food and culture. Cooking was addressed as an enjoyable activity that brings people together, creates bonds, establishes trust, promotes socialization, and provides a healthy meal to those in need. |
| 5 | Dating | Group-based activities and creating a dating site were considered to have potential benefits for finding meaningful and strong relationships, which can lead to improved overall mental health, self-esteem, relaxation, sexual health, societal integration, and reduced loneliness. It was considered challenging for forced migrants with diverse SOGIE to date and find a partner, involving a risk of encountering abuse, unhealthy habits, contracting sexually transmitted infections, and feeling disappointed. A related suggested intervention involved health education about consent, dating, and safe sex practices. |
| 6 | Games and sports | Group-based activities were considered to have benefits for physical and mental health, including sleep, mental focus, stress, anxiety, and happiness. Participating in games and sports were addressed as bringing people together and helping them find meaningful company, while also promoting them to gain healthy habits and feel freedom, pleasure, optimism, and creativeness. |
